# Revisiting PPARγ as a new friend of GPR120 in the treatment of metabolic disorders

**DOI:** 10.1080/21623945.2020.1838186

**Published:** 2020-10-27

**Authors:** Vivian A. Paschoal, Da Young Oh

**Affiliations:** Touchstone Diabetes Center, Department of Internal Medicine, University of Texas Southwestern Medical Center, Dallas, TX, USA

**Keywords:** GPR120, PPAR gamma, type 2 diabetes, insulin resistance, metabolism

## Abstract

G Protein-coupled receptor 120 (GPR120; fatty acid receptor 4, FFAR4) and PPARγ agonists both lead to anti-inflammatory and insulin sensitizing effects despite signalling through distinct pathways. We recently reported the overarching idea that these two pathways are interactive. Specifically, treatment of obese mice with the PPARγ agonist rosiglitazone (a thiazolidinedione, TZD) in combination with the GPR120 agonist compound A synergistically improves glucose tolerance and insulin sensitivity. We have deconvoluted the mechanisms underlying this feed-forward effect in the study. Taken together, our study shows that low dose TZD administration, in combination with GPR120 agonists, produces additive beneficial effects on glucose tolerance and insulin sensitivity without the undesirable adverse effects of TZD. Our study suggests potential value of combination PPARγ and GPR120 agonists to treat metabolic disease.

The discovery that adipose tissue of obese mice and humans is infiltrated with macrophages provided a major mechanistic advance in our understanding of how obesity promotes inflammation [1,2]. Macrophages are a major source of proinflammatory cytokines that can decrease insulin sensitivity through a paracrine and potentially an endocrine mechanism. Over the past decade, we and many other groups have assessed the pathogenic role of macrophages as an initiator of insulin resistance. Specifically, we found that G protein-coupled receptor 120 (GPR120), upon activation by its ligand omega-3 fatty acids (ω3-FAs, including DHA and EPA) produce robust anti-inflammatory, insulin sensitizing effects both *in vivo* and *in vitro* [3]. Since the large amount of ω3-FAs that would have to be consumed to sustain GPR120 activation is not practical, various high-affinity small molecule GPR120 agonists have been developed for potential clinical use by the pharmaceutical industry. We have identified a high-affinity, selective GPR120 agonist (compound A; CpdA) that exerts potent anti-inflammatory effects on macrophages *in vitro* and in obese mice in a GPR120 dependent manner [4]. GPR120 agonist treatment of HFD/obese mice enhanced anti-inflammatory signalling, glucose tolerance, and insulin sensitivity while decreasing hyperinsulinemia and hepatic steatosis [3,4]. This suggests that GPR120 agonists could emerge as a new insulin-sensitizing agent for T2D treatment.

TZDs (thiazolidinedione), such as rosiglitazone and pioglitazone, are effective therapeutic agents for insulin resistance in humans. However, they can cause adverse effects such as oedema [5–7], weight gain [8,9], heart failure [10–12], bone loss [13,14], and apparently certain cancers [15,16]. While the exact mechanisms of TZD-induced insulin sensitization are not fully understood, these agents act through PPARγ, which has been extensively reviewed elsewhere [17–19]. Therefore, downstream targets of PPARγ are believed to mediate insulin sensitization.

The key idea of our recent paper [20] is that TZDs cause insulin sensitization by acting through PPARγ and its target genes. In addition, GPR120 agonists cause anti-inflammation and insulin sensitivity through a GPR120 signalling pathway that involves β arrestin-2 and G_q/11_ coupling. Since these two classes of agonists cause insulin sensitization through distinct mechanisms, we wondered if their co-administration may provide an additive effect. Our hyperinsulinemic-euglycemic clamp studies showed that in HFD-fed mice, the insulin-sensitizing effects of rosiglitazone (Rosi) on WT mice was substantially attenuated in the GPR120 KO mice. This was the first clue that the two pathways interact. We then set out to do multiple treatment studies at different doses of rosiglitazone to assess the potential of additivity with GPR120 agonist *in vivo*. Using the GPR120 agonist compound A (CpdA), we see a robust additivity when combined with Rosi treatment in HFD-fed WT mice but not in GPR120 KO mice. Importantly, we established the lowest dose of Rosi that gives a detectable albeit weak improvement of glucose tolerance and insulin sensitivity. When this ‘minimal’ dose of Rosi is co-administered with a GPR120 agonist, we get full insulin sensitization and improved glucose tolerance much like ‘maximal’ dose of Rosi. This demonstrates the additivity of these mechanisms.

To address the mechanisms for this additivity, we conducted detailed *in vivo* physiologic as well as cellular and molecular studies. From the physiologic point of view, we generated adipocyte (AKO) and macrophage specific (MKO) GPR120 knockout models. These two models, along with other *in vivo* data using a GPR120 agonist (CpdA treatment) enabled us to identify an *in vivo* mechanistic basis for the combined effects of PPARγ and GPR120 agonists. The tissue-specific KO mouse models demonstrate the concept that the insulin sensitizing effects of the GPR120 agonists are primarily mediated through their antiinflammatory actions in macrophages while adipocytes play a minor role. This is contrast to Rosi, which acts primarily on adipocytes. In other words, the additivity *in vivo* from a whole system point of view arises by complementing the effect of CpdA on macrophages with that of Rosi on adipocytes (Figure 1). We also found that CpdA has direct insulin sensitizing effects in adipocytes, resulting in an additional layer of additivity from a cell autonomous point of view.

**Figure 1.**
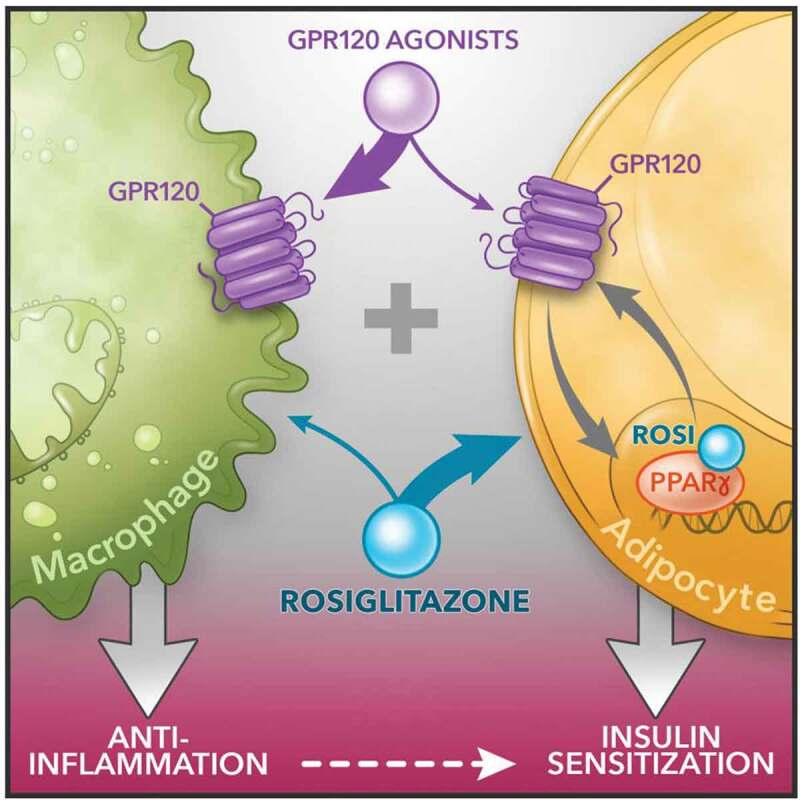
Schematic diagram of dual agonism of GPR120 and PPARγ to improve insulin resistance. Figure is generated by Richard Howdy from Visually Medical

Our studies show that GPR120 is a PPARγ target gene, and its induction by Rosi enables TZDs to potentiate the effects of GPR120 agonist. Conversely, the GPR120 agonist stimulates the production of 15d-PGJ2, which we established as an endogenous PPARγ agonist in adipocytes that stimulates the transactivation of PPARγ target genes including GPR120, providing another layer of feed-forward, and NCoR dissociation, CDK5 dissociation, etc. Although 15d-PGJ2 can clearly stimulate PPARγ, its physiological and pharmacological relevance has been debated [21,22]. For example, the concentrations required to activate PPARγ are generally reported in the μM range, while many other endogenous prostaglandins are effectively act at low nM concentrations. Consistent with this, we were not able to detect 15d-PGJ2 in mouse plasma. However, 15d-PGJ2 acts intracellularly to activate PPARγ, in contrast to other prostaglandins that principally act as extracellular ligands of cell surface receptors. Thus, future studies on the role of 15d-PGJ2 as an endogenous PPARγ activator will be of interest particularly related to bioactive intracellular and microenvironmental concentrations. As an additional mechanism, stimulation of GPR120 with CpdA maintains PPARγ S273 in the unphosphorylated state [20], which is more active than the phosphorylated form [23–25]. The mechanism for this is that in response to extracellular CpdA, cell-surface GPR120 recruits β arrestin-2, leading to endocytic internalization of the two proteins. The internalized β arrestin-2 in turn colocalizes with and sequesters ERK (the main kinase for S273) [23] in the cytosol, preventing its nuclear translocation and phosphorylation of PPARγ S273. Together these cellular mechanisms outline a positive reinforcing interaction between these two signalling systems within adipocytes.

In summary, the overall additivity is explained by overarching mechanism (Figure 1):

1. *In vivo*, Effects on macrophages (GPR120) and adipocytes (GPR120 + PPARγ) combining to cause enhanced insulin sensitivity.
2. Adipocyte-autonomous additivity between GPR120 and PPARγ signalling.

Beyond enhancing therapeutic efficacy, combination treatment may also provide the benefit of minimizing side effects. Specifically, although TZDs have been FDA approved for many years, their clinical use is compromised by well-known side effects particularly weight gain and oedema. We find that combination treatment with GPR120 agonists and Rosi prevents the effects of Rosi to cause weight gain and oedema, even if we used maximal dose of Rosi. We do not know the mechanisms for this, but they are consistent across many cohorts of mice. Furthermore, because we find additive combinatorial efficacy on insulin sensitivity, we were able to titrate down Rosi to a ‘minimal’ dose that does not lead to body weight gain or oedema.

Whether this benefit extends to other side effects of TZDs like bone loss is currently unknown.

Taken together, we propose that simultaneous targeting of GPR120 and PPARγ may have clinical value. The mechanisms we defined to provide a compelling explanation for the *in vivo* additivity, which leads to greater efficacy and less side effects.
